# Design and Fabrication of Posture Sensing and Damage Evaluating System for Underwater Pipelines

**DOI:** 10.3390/s25185927

**Published:** 2025-09-22

**Authors:** Sheng-Chih Shen, Yung-Chao Huang, Chih-Chieh Chao, Ling Lin, Zhen-Yu Tu

**Affiliations:** Department of Systems and Naval Mechatronic Engineering, National Cheng Kung University, Tainan City 701, Taiwan; p18141041@gs.ncku.edu.tw (Y.-C.H.); p18141025@gs.ncku.edu.tw (C.-C.C.); p16134072@gs.ncku.edu.tw (L.L.); p16114022@gs.ncku.edu.tw (Z.-Y.T.)

**Keywords:** underwater pipeline, Hall sensor, inertial sensor module, early warning system, posture algorithm

## Abstract

This study constructed an integrated underwater pipeline monitoring system, which combines pipeline posture sensing modules and pipeline leakage detection modules. The proposed system can achieve the real-time monitoring of pipeline posture and the comprehensive assessment of pipeline damage. By deploying pipeline posture sensing and leakage detection modules in array configurations along an underwater pipeline, information related to pipeline posture and flow variations is continuously collected. An array of inertial sensor nodes that form the pipeline posture sensing system is used for real-time pipeline posture monitoring. The system measures underwater motion signals and obtains bending and buckling postures using posture algorithms. Pipeline leakage is evaluated using flow and water temperature data from Hall sensors deployed at each node, assessing pipeline health while estimating the location and area of pipeline damage based on the flow values along the nodes. The human–machine interface designed in this study for underwater pipelines supports automated monitoring and alert functions, so as to provide early warnings for pipeline postures and the analysis of damage locations before water supply abnormalities occur in the pipelines. Underwater experiments validated that this system can precisely capture real-time postures and damage locations of pipelines using sensing modules. By taking flow changes at these locations into consideration, the damage area with an error margin was estimated. In the experiments, the damage areas were 8.04 cm^2^ to 25.96 cm^2^, the estimated results were close to the actual area trends (R^2^ = 0.9425), and the area error was within 5.16 cm^2^ (with an error percentage ranging from −20% to 26%). The findings of this study contribute to the management efficiency of underwater pipelines, enabling more timely maintenance while effectively reducing the risk of water supply interruption due to pipeline damage.

## 1. Introduction

With increased demand for ocean resource development, deep seawater, characterized by low temperature, purity, and mineral richness, is widely used across industries such as health foods, aquaculture, and health tourism. Coastal areas with steep seabed topography are ideal for deep seawater extraction, and subsea pipelines serve as a critical link in ensuring stable deep seawater intake. However, these pipelines are frequently exposed to damage risk, including buckling, fracture, displacement, and slippage of ballast blocks, caused by natural disasters such as typhoons, earthquakes, and long swells. Such damage severely threatens water supply stability and industrial growth [1,2]. 

Currently, various pipeline monitoring technologies exist in both the industry and academia, utilizing principles from optical, acoustic, magnetic, and electrical fields. For instance, fiber optic sensors can detect minor strains in pipelines [3]; ultrasonic transmission and echo analysis can identify internal defects through acoustic reflections [4,5]; magnetic flux leakage testing [6,7] and eddy current scanning [8] are specialized in detecting metal loss and surface flaws; hydrophones can monitor acoustic signals generated by pipeline leaks [9]. Furthermore, autonomous underwater vehicles (AUVs) equipped with multiple sensing modules and pipeline inspection robots are extensively utilized for routine inspection tasks [10].

In addition to the aforementioned physical sensing technologies, data-driven pipeline monitoring methods have demonstrated substantial potential in recent years. Notably, by integrating high-precision pressure and flow sensors alongside advanced algorithms such as neuro-fuzzy banded Kalman filters (ZKF), these methods can accurately identify nonlinear dynamics in pipelines, and effectively detect sensor failures and leaks. Such methods require only fault-free data for training and can handle common parameter uncertainties and measurement noise in hydraulic pipelines. Experimental results indicate that these methods can achieve an accuracy of up to 99.24% in leak detection, highlighting their exceptional efficacy in enhancing monitoring reliability [11].

However, despite demonstrating specific detection capabilities and application potentials, the full-scale practical deployment of these inspection technologies in specific scenarios such as deep seawater intake systems remains constrained. While these technologies are well-established for industrial or terrestrial pipelines, their application in deep-sea environments introduces unique challenges. For example, some optical and magnetic sensors require specialized underwater connectors and sealing to withstand high-pressure, which significantly increases costs and maintenance complexity. Similarly, acoustic and electrical methods can suffer from signal interference or attenuation due to high water pressure and temperature fluctuations. This necessitates robust equipment and specialized operation, often limiting their efficiency and accuracy compared to terrestrial use. Consequently, the adoption of these technologies for deep-sea pipelines is often limited by high costs, logistical complexities, and the need for specialized deployment [12,13]. To provide a clearer comparison of the advantages and limitations of each technology, the performances of these technologies were analyzed, as shown in Table 1.

This study aimed to integrate Hall flow sensing modules with inertial measurement modules, in order to address challenges of monitoring deep seawater intake facilities. It employed posture estimation and damage prediction algorithms to develop a pipeline monitoring system that can achieve the real-time monitoring of pipeline posture and flow fluctuations. It also applied the Computational Fluid Dynamics (CFD) theory to infer the locations and areas of pipeline damage. The system integrates a human–machine interface and warning module, equipped with real-time abnormality reporting functions, visual monitoring, and historical data tracking, in order to enhance maintenance efficiency and response speed. Compared to existing high-cost inspection methods, the proposed system offers the advantages of simple installation, low cost, and suitability for long-term continuous monitoring, demonstrating application potential for monitoring various underwater structures.

## 2. Methods

**Figure 1:**
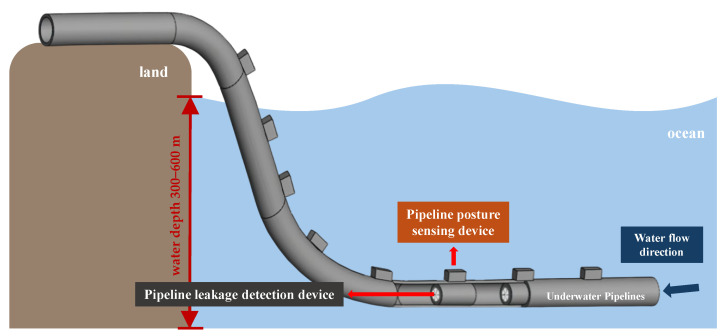
Schematic diagram of the configuration of subsea sensor modules in the pipeline system.

This study integrated the pipeline posture sensing and leakage detection modules to establish the subsea sensing module, facilitating the development of an underwater pipeline monitoring system. The subsea sensing modules were deployed in arrays along the external surface and the internal bore of the pipeline, enabling real-time underwater monitoring (Figure 1). The posture sensing modules were used to collect the motion posture of the pipeline. In contrast, the pipeline leakage detection modules were used to analyze the damaged area and identify leak locations based on the CFD theory.

**Figure 2:**
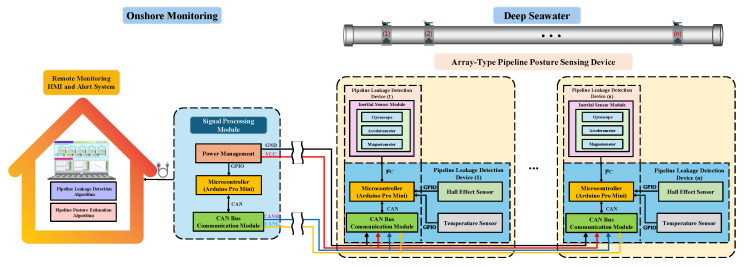
Schematic diagram of the signal transmission architecture for underwater sensing modules.

This system measured the posture variation of a subsea water intake pipeline. It comprises underwater sensing modules and the monitoring interface device, as shown in Figure 2. Signal data transmission was conducted using a Controller Area Network (CAN Bus) to ensure the real-time delivery and stability of monitoring information transmission. In underwater sensing parts, the pipeline posture sensing modules are used to monitor pipeline postures. When deviations in the pipeline posture occur, the integrated accelerometer, gyroscope, and magnetometer can provide nine different physical parameters. These can be integrated with the Extended Kalman Filter (EKF) to enhance the algorithm’s accuracy. The pipeline leakage detection module monitors leak locations and estimates the extent of the damage area. Using Hall sensors equipped with internal impellers and magnetic sensing units, the module can detect the corresponding Hall effect signals generated by the rotation of the impellers caused by water flow. Signals are converted to frequency output, allowing for the measurement of flow rates based on frequency variations. When abnormal variations in flow rates are detected, leakage locations and the corresponding area of damage can be estimated.

### 2.1. Pipeline Posture Sensing Module

**Figure 3:**
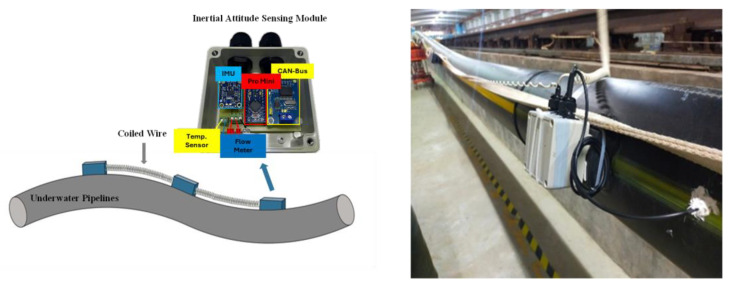
Pipeline posture sensing module and photograph of the actual pipeline configuration diagram.

The pipeline posture sensing module integrates the microcontroller, the CAN Bus module, and the inertial sensor module. Its configuration along the pipeline is presented in Figure 3. The inertial sensor module includes a three-axis accelerometer, gyroscope, and magnetometer, which can collect the acceleration signals, angular velocity signals, and magnetic signals of the pipeline’s subsea postures, estimating the bending, buckling, and other abnormal pipeline states. All data collected by the sensing modules along the pipeline were transmitted via the CAN Bus network to the pipeline monitoring system for real-time computation and processing of the pipeline’s posture. The CAN Bus utilizes differential signaling to reduce electromagnetic interference and it features an auto-retransmission mechanism, ensuring stable and reliable remote monitoring. Furthermore, extendable coiled connections between sensors can minimize cable strain caused by posture changes, enhancing the reliability of the posture sensing module.

**Figure 4:**
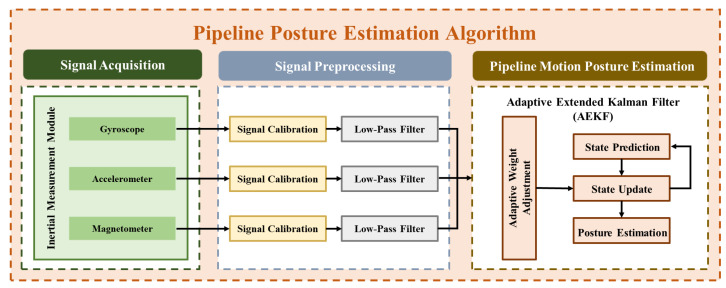
Pipeline posture estimation algorithm flowchart.

The workflow of the pipeline posture estimation algorithm is presented in Figure 4. First, the pipeline posture sensing module captures the pipeline’s subsea motion signals. To minimize errors arising from intrinsic sensor properties and extrinsic environmental factors, preliminary signal calibration of the pipeline posture sensor is essential. Since turbulence and pipeline drift due to ocean currents may introduce high-frequency noise, noise filtering is designed to remove such high-frequency noise from motion posture signals. Next, the pipeline’s real-time posture angles can be calculated based on motion posture estimation. Finally, array-type sensing modules along the pipeline continuously record changes in posture angles, providing information such as motion angles, bending angles, and buckling angles. Next, the posture estimation algorithm can be used to estimate the pipeline bending posture.

#### Posture Estimation Algorithm

This study utilized the pipeline posture sensing module to collect the pipeline’s subsea posture signals. Sensing modules separately recorded dynamic information regarding acceleration, angular velocity, and magnetic field strength as for the pipeline posture estimation. However, due to intrinsic sensor properties and extrinsic environmental factors, the signals measured by the accelerometer, gyroscope, and magnetometer are susceptible to signal drift. Over time, drift accumulates, leading to cumulative errors in pipeline posture estimation after the integral computation. Therefore, this study adopted signal preprocessing methods to reduce cumulative errors and obtain posture data with higher accuracy. The signal preprocessing methods include the following: (1) Signal calibration technologies. To calibrate accelerometer and gyroscope signals, sensors were installed in a 20 °C incubator; a multi-directional rotation device was used to record output signals under various orientations, thereby determining scale factors and offsets for calibration. Magnetometer calibration involves three-axis rotation in an environment free of strong magnetic interference, using the least squares method for matrix calibration to obtain the calibrated magnetometer outputs; (2) Noise filtering technologies. Since sensors are affected by high-frequency noise such as ocean currents in the marine environment, low-pass filters are used to filter out these interferences and obtain clear motion posture signals. Once filtered, the calibrated acceleration signal $a_{b}\;=\;{\left[a_{bx},a_{by},a_{bz}\right]}^{T}$, angular velocity signal $\omega_{b}\;=\;{\left[\omega_{bx},\omega_{by},\omega_{bz}\right]}^{T}$, and magnetic field signal $m_{b}\;=\;{\left[m_{bx},m_{by},m_{bz}\right]}^{T}$ can be obtained. For estimating the pipeline’s posture angles, nine calibrated sensing signals were input into the EKF, fusing the acceleration, angular velocity, and magnetic field data to enhance pipeline posture estimation accuracy. Acceleration and magnetic values may be affected by the motion acceleration of the external pipeline movements and ambient magnetic field interference, leading to posture estimation errors. Therefore, the Adaptive Weighting Adjustment parameters within the EKF were adjusted to reduce the instant response time of the accelerometer and magnetometer under external interference, improving the stability of posture estimation. The algorithm is based on the following steps:

1. State prediction: The posture expressed in quaternions is defined as the state variable (x) in the state transition equation, establishing a state transition model using the angular velocity ($\omega_{t}$) measured by the gyroscope at the current time (t) and the updated posture angle ($x_{t-1}^{\text{’}}$) from the previous time point (t − 1), as shown in Equation (1):
  (1) $${\hat{x}}_{t}=A_{t}x_{t-1}^{\prime }+B_{t-1}\delta \omega_{bt}$$
  where ${\hat{x}}_{t}\;=\;{\left[{\hat{q}}_{0,t},{\hat{q}}_{1,t},{\hat{q}}_{2,t},{\hat{q}}_{3,t}\right]}^{T}$ denotes the predicted state at the current time. $A_{t}$ $I_{4\text{×}4}+\frac{1}{2}\Omega \left(\omega_{t}\right)\Delta t$ represents the state transition matrix at the current time. $B_{t-1}$$\;=\;\frac{\text{∂}A_{t}x_{t-1}^{\text{‘}}}{\text{∂}\delta \omega_{t}}\;=\;\frac{1}{2}\text{∆}t\left[\begin{array}{ccc} {-q}_{1,t-1}^{\text{‘}} & {-q}_{2,t-1}^{\text{‘}} & {-q}_{3,t-1}^{\text{‘}}\\ q_{0,t-1}^{\text{‘}} & -q_{3,t-1}^{\text{‘}} & q_{2,t-1}^{\text{‘}}\\ q_{3,t-1}^{\text{‘}} & q_{0,t-1}^{\text{‘}} & {-q}_{1,t-1}^{\text{‘}}\\ {-q}_{2,t-1}^{\text{‘}} & q_{1,t-1}^{\text{‘}} & q_{0,t-1}^{\text{‘}} \end{array}\right]$ denotes the process noise coefficient matrix from the previous time point., ${\delta \omega }_{t}\;=\;{\left[{\delta \omega }_{x,t},\delta \omega_{y,t},\delta \omega_{y,t}\right]}^{T}$ denotes the white noise of angular velocity at the current time, $I_{4\text{×}4}$ denotes 4 × 4 a unit matrix, $\omega_{t}\;=\;{\left[\omega_{x,t},\omega_{y,t},\omega_{z,t}\right]}^{T}$ denotes the angular velocity sensed by the gyroscope at the current time, while $\Omega \left(\omega_{t}\right)\;=\;\left[\begin{array}{cccc} 0 & -\omega_{x,t} & -\omega_{y,t} & -\omega_{z,t}\\ \omega_{x,t} & 0 & \omega_{z,t} & -\omega_{y,t}\\ \omega_{y,t} & -\omega_{z,t} & 0 & \omega_{x,t}\\ \omega_{z,t} & \omega_{y,t} & -\omega_{x,t} & 0 \end{array}\right]$, and Δt is the sampling period.

The prediction for the state covariance matrix $\left({\hat{P}}_{t}\right)$ is shown in Equation (2):

(2) $${\hat{P}}_{t}=A_{t}P_{t-1}^{\prime }A_{t}^{T}+B_{t-1}QB_{t-1}^{T}$$

where $P_{t-1}^{\prime }$ represents the state error covariance matrix updated at the previous time step. $Q=E\left[\delta \omega_{bt}\delta {\omega_{bt}}^{T}\right]$ represents the angular velocity noise covariance matrix.

2. Adaptive weight adjustment: Through the adaptive adjustment of the acceleration noise covariance matrix ($R_{a}$) and the magnetic noise covariance matrix ($R_{m}$) during state update, the weight of the Kalman gain (K) can be adjusted. This shortens the filter’s transient response time and enhances the stability of the pipeline posture estimation. First, errors in the acceleration and magnetic values due to motion acceleration or magnetic disturbances were divided into magnitude errors ($E_{m,t}$) and direction errors ($E_{d,t}$), as shown in Equations (3) and (4). Next, magnitude and direction errors were synthesized using a natural exponential function to obtain the adaptive trust parameter $r_{c}$. Next, Equation (5) shows that the parameter reflects the reliability of the acceleration or magnetic values under external disturbances: as $r_{c}$ approaches 0, the reliability of using acceleration and magnetic values to estimate postures decreases, indicating significant external influences on the inertial sensors, as $r_{c}$ approaches 1, the reliability of using the acceleration and magnetic values to estimate the postures increases, indicating minimal external influences on the inertial sensors. Finally, the adaptive trust parameter $r_{c}$ was used to adjust the acceleration or magnetic noise covariance matrices, achieving optimized Kalman gain. This serves to reduce the EKF’s instantaneous response time and improve the stability of the overall posture estimation process.
  (3) $$E_{m,t}=max\left(\frac{s_{m,t}}{r_{m}},\frac{r_{m}}{s_{m,t}}\right)\in \left[1,+\infty \right)$$
  (4) $$E_{d,t}=\left\|{\left(R_{i,t}^{r}\right)}^{T}r_{d}-s_{d,t}\right\|\in \left[0,2\right]$$
  where $s_{m,t}$ is the resultant acceleration value ($s_{m,t}=\left\|a_{i,t}\right\|$), measured by the accelerometer or the resultant magnetic value, measured by the magnetometer at the present time. $r_{m}$ is the amplitude intensity of gravity acceleration, measured by the accelerometer when it is at rest or the intensity of the Earth’s magnetic amplitude field without magnetic field interference. $s_{d,t}$ is the gravity unit vector $\left(s_{d,t}=\frac{a_{i,t}}{\left\|a_{i,t}\right\|}\right)$, measured by the accelerometer or the unit vector of magnetic north, measured by the magnetometer at the present time. $r_{d}$ is the gravity unit vector ${(r}_{d}={\left[0,0,1\right]}^{T})$ or the unit vector of magnetic north ${(r}_{d}={\left[0,1,0\right]}^{T})$ in the reference coordinate system.
3. State update: The errors produced in state prediction from angular velocity may accumulate over time. Therefore, it is essential to update the predicted state using the observations ($z_{t}$). This paper defines the observations as the acceleration and magnetic force values, and establishes a gravitational or magnetic north observation equation to update the posture state (${\hat{x}}_{t}$) predicted by angular velocity, as shown in Equation (5).
  (5) $$z_{t}^{h}={H_{t}\hat{x}}_{t}-{\delta z}_{t}$$
  where $H_{t}$ is the gravitational observation matrix or the magnetic north observation matrix at the current time, ${\hat{x}}_{t}={\left[{\hat{q}}_{0,t},{\hat{q}}_{1,t},{\hat{q}}_{2,t},{\hat{q}}_{3,t}\right]}^{T}$ denotes the predicted state variable at the current time, $\delta z_{t}$ is the white noise of the acceleration or magnetic force value at the current time, and $z_{t}^{h}$ is the predicted observation value at the current time. Following the acquisition of the gravitational or magnetic north observation equation, the updated Kalman gain ($K_{t}$) for the gravity or magnetic north at the current time is calculated, as shown in Equation (6). Finally, the updated Kalman gain for gravity or magnetic north is utilized to update the predicted state and its state error covariance matrix, as shown in Equations (7) and (8).
  (6) $$K_{t}={\hat{P}}_{t}H_{t}^{T}{\left({H_{t}{\hat{P}}_{t}H}_{t}^{T}+R\right)}^{-1}$$
  (7) $$x_{t}^{\prime }={\hat{x}}_{t}+K_{t}\left(z_{t}-z_{t}^{h}\right)$$
  (8) $$P_{t}^{\prime }=\left(I_{4\times 4}-K_{t}H_{t}\right){\hat{P}}_{t}$$
  where $R\;=\;E\left[\delta z\delta \mathrm{zT}\right]$ denotes the observation noise covariance matrix; when the observation is acceleration, R is the gravity noise covariance matrix $\left(R_{a}\right)$. However, when the observation is the magnetic value, R is the magnetic north noise covariance matrix $\left(R_{m}\right);\;z_{t}\;$ is the observation value of gravity or magnetic north measured at the current time.

After completing the above calculation steps, the posture angle, bending angle, and buckling angle of the intake pipeline can be obtained, as shown in Equations (9)–(11). With this method, the bending and buckling angles of the pipeline can be calculated, providing the posture estimation results.

(9) $$\phi =\tan^{-1}\left(\frac{2\left({\hat{q}}_{2}^{\prime }{\hat{q}}_{3}^{\prime }+{\hat{q}}_{0}^{\prime }{\hat{q}}_{1}^{\prime }\right)}{{{\hat{q}}_{0}^{\prime }}^{2}-{{\hat{q}}_{1}^{\prime }}^{2}-{{\hat{q}}_{2}^{\prime }}^{2}+{{\hat{q}}_{3}^{\prime }}^{2}}\right)$$

(10) $$\theta =\sin^{-1}\left(-2{(\hat{q}}_{1}^{\prime }{\hat{q}}_{3}^{\prime }-{\hat{q}}_{0}^{\prime }{\hat{q}}_{2}^{\prime })\right)$$

(11) $$\psi =\tan^{-1}\left(\frac{2\left({\hat{q}}_{1}^{\prime }{\hat{q}}_{2}^{\prime }+{\hat{q}}_{0}^{\prime }{\hat{q}}_{3}^{\prime }\right)}{{{\hat{q}}_{0}^{\prime }}^{2}+{{\hat{q}}_{1}^{\prime }}^{2}-{{\hat{q}}_{2}^{\prime }}^{2}-{{\hat{q}}_{3}^{\prime }}^{2}}\right)$$

**Figure 5:**
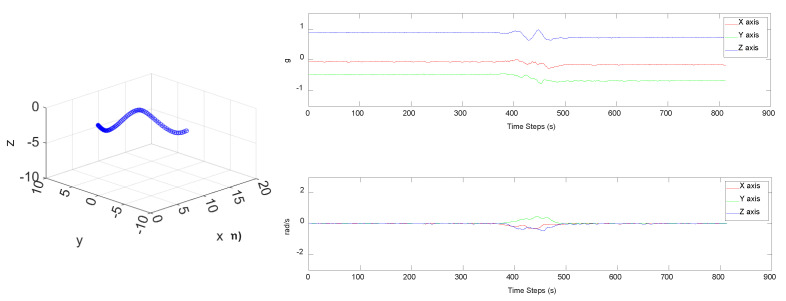
Pipeline posture profile of the pipeline in a W-shaped configuration.

In this study, the pipeline posture sensing module provides signals from the accelerometer, gyroscope, and magnetometer. The resulting nine-axis signals were processed via the posture estimation algorithm to derive motion posture parameters. Then, acceleration vectors were transformed into acceleration relative to the Earth’s axis, allowing for the calculation of velocity and displacement to output a posture model. Figure 5 shows the real-time posture of the pipeline in a “W” configuration, as detected by the pipeline posture sensing module. It validates the EKF-based posture algorithm’s capability to accurately estimate the real-time shape of the pipeline posture.

### 2.2. Pipeline Leakage Detection Module

The pipeline leakage detection module integrates the microcontroller, the CAN Bus module, the Hall sensors, and the temperature sensors to measure the flow rate and temperature within the deep-sea water intake pipeline. It detects pipeline damage and anomalies and estimates the location and extent of the damage to the pipeline. Within the leakage detection module, the microcontroller receives signals from the Hall sensors and thermometers installed inside the pipeline. Data are transmitted via the CAN Bus module to the pipeline monitoring system. After computation, sensing signals are displayed on the human–machine interface, achieving real-time monitoring of flow rate variations. Pipeline leakage detection modules were deployed every 3.5 m along the entire pipeline.

The damage detection algorithm is based on an intuitive and effective logic: continuous monitoring of the flow variations between adjacent Hall sensors. According to fluid dynamics principles, when the pipeline is intact, the flow readings of the preceding and following sensors should be consistent or exhibit only minor fluctuations. However, once damage occurs in the pipeline, as external pressure exceeds internal pressure, seawater flows into the pipeline from the damaged areas, thereby leading to a significant increase in flow readings at the downstream sensors. For instance, when the downstream sensor’s reading increases relative to its upstream counterpart and surpasses a predetermined threshold (e.g., 10 L/min), the system promptly identifies damage in that interval. This logic is evident, as the abnormal increase in flow directly indicates the intrusion of external water.

**Figure 6:**
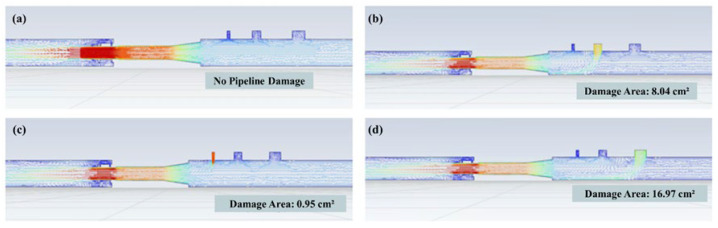
Pipeline structure and configuration; three types of damage holes in the pipeline. (**a**) No pipeline damage. (**b**) 8.04 cm^2^ damage area. (**c**) 0.95 cm^2^ damage area. (**d**) 16.97 cm^2^ damage area.

**Figure 7:**
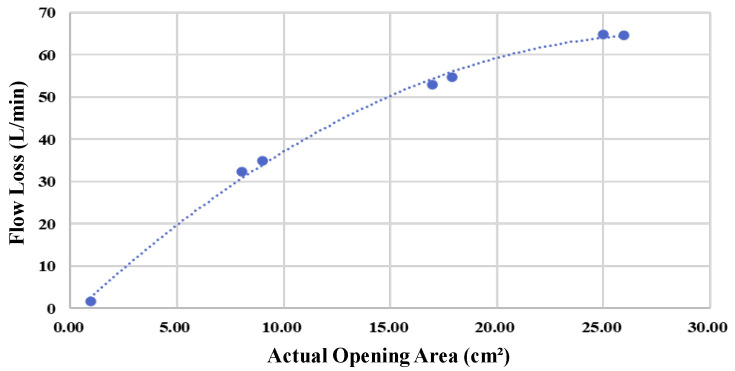
Relationship between pipeline damage area and lost flow.

The CFD theory can be used to visualize the internal flow field distribution, pressure variations, and velocity characteristics of the pipeline. It can also assess the influence of damage on the pipeline’s flow patterns, thus predicting flow velocity distribution, pressure changes, and regions exhibiting eddy or turbulent flows inside the pipeline. This study used CFD to simulate the subsea pipeline configuration.(This study used CFD to simulate the subsea pipeline configuration with SOLIDWORKS 2018 Flow Simulation.) The pipeline model includes the primary structure, the internal converging nozzle design, and the deployment and distance of Hall sensors, obtaining the analysis results regarding fluctuations in flow and the area of damage when the pipeline is damaged. The pipeline was modeled with a total length of 20 m and an external diameter of 12.5 cm. A converging nozzle was designed inside the pipeline with an inlet diameter of 10 cm. This was gradually reduced to 5 cm to simulate the influence of diameter changes on flow fields. To explore the pipeline fluid dynamics under damage, three damage holes of varying sizes—0.95 cm^2^, 8.04 cm^2^, and 16.97 cm^2^—were designed on the pipeline surface (Figure 6). The boundary conditions set the pipeline at a position 2 m below the surface with corresponding hydrostatic pressure, a pipeline inlet pressure of 220 kPa, a water flow rate of 420 L/min, and an outlet flow velocity of 0.66 m/s. This served to analyze the relationship between different pipeline damage areas and flow variations (Figure 7).

The simulation results depict fluid velocity using a color gradient from blue (slow) to red (fast). When the pipeline is intact, as in Figure 6a, the fluid flows uniformly inside the pipeline, with no evidence of leakage or other anomalies. Fluid velocity remains roughly constant throughout, with more pronounced changes occurring only near the inlet and outlet regions. Particularly in the pipeline’s narrow sections, a reduction in cross-sectional area leads to increased fluid velocity. The flow within the pipeline is stable and exhibits a predictable velocity distribution. Figure 6b–d shows that when the pipeline is damaged, external fluid enters the pipeline primarily due to the differential pressure (internal pressure is lower than external pressure). Therefore, the flow velocity manifests as a transition from blue (slow flow velocity) to red (high flow velocity). Furthermore, a marked difference in flow signals is detected by the Hall sensors installed before and after the pipeline’s damage locations. Figure 7 shows that the hole with an area of 5 cm^2^ resulted in a 20 L/min flow variation; the hole with an area of 10 cm^2^ resulted in a 37 L/min flow variation; the hole with an area of 15 cm^2^ resulted in a 50 L/min flow variation. The relationship between pipeline damage area and flow variation serves as a critical reference for the accurate estimation of the pipeline’s damage area and location.

The monitoring system designed in this study can effectively distinguish between transient flow anomalies and actual damage to the pipeline structure. This can be attributed to the physical characteristics tailored to the specific application context of deep seawater intake pipelines. Under normal operational conditions, the fluid pressure within the pipeline is less than the external hydrostatic pressure at the corresponding water depth. Based on this characteristic, the system employs sensors arranged in a multi-point array within the pipeline, so that the multidimensional data correlation and assessments can be conducted. Specifically, if the flow reading from a single sensor significantly increases, and the downstream sensor’s flow reading also rises, it indicates actual damage in the pipeline, thus resulting in the flow of seawater into the pipeline. Conversely, if the flow reading from a single sensor decreases, the system can further examine data from the posture sensing module. Under this condition, if abnormal bending of the pipeline is detected, it may indicate a pipeline blockage caused by structural damage. Furthermore, the system can compare the flow values from upstream sensors. If the readings of upstream sensors remain normal or increase, this further supports the indication of a blockage. This comprehensive data correlation analysis enables the system to effectively identify and eliminate flow anomalies caused by transient disturbances, thus facilitating accurate diagnosis of pipeline damages or structural failures.

## 3. Results

**Figure 8:**
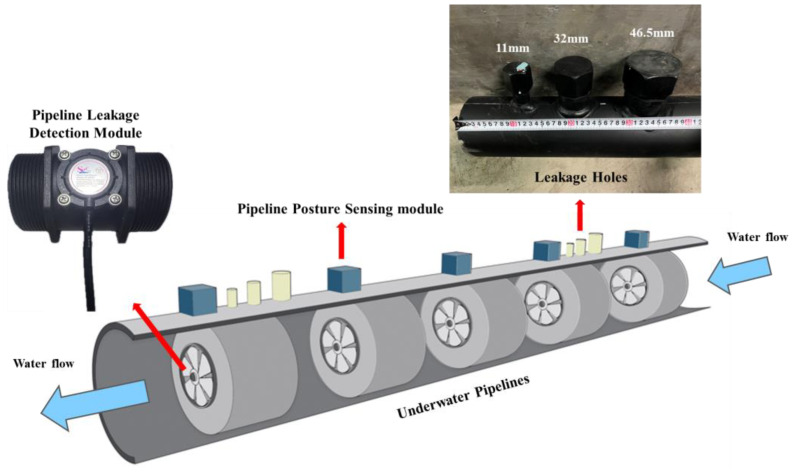
Pipeline configuration diagram.

This study conducted underwater experiments for pipeline posture variation and pipeline leakage in the towing tank of the Department of Systems and Naval Mechatronic Engineering, National Cheng Kung University (NCKU Towing Tank). The towing tank measures 330 m in length, 8 m in width, and 4 to 5.5 m in depth. It is equipped with a wave maker capable of producing regular waves of varying frequencies to simulate different subsea conditions for the comprehensive testing of underwater pipelines. The subsea intake pipeline examined in this study was fabricated from high-density polyethylene (HDPE), with a length of 20 m and an outer diameter of 12.5 cm. Pipeline posture sensing modules and pipeline leakage detection modules were installed at 3.5 m intervals along the pipeline. The sensor configuration is illustrated in Figure 8. For pipeline posture estimation experiments, arrays of posture sensing modules were distributed along the pipeline’s surface to measure underwater motion signals. Inertial signals collected at each node were processed using a posture estimation algorithm to estimate the pipeline’s bending and buckling states. To validate the estimation capabilities of the pipeline leakage detection module for the pipeline leakage area and flow variation in the pipeline leakage estimation experiments, three holes of different diameters were designed between the second and fifth segments of the pipeline. The flow rates measured by Hall sensors at each node were used to estimate the location and size of pipeline damage, allowing for the monitoring of damage along the intake pipeline.

### 3.1. Seawater Pipeline Posture Observation Experiments

**Figure 9:**
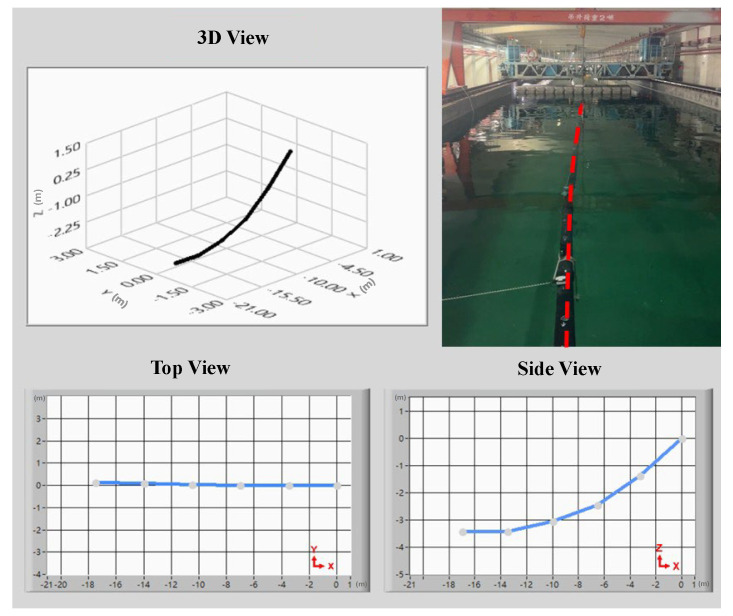
Vertical submersion of the seawater pipeline.

**Figure 10:**
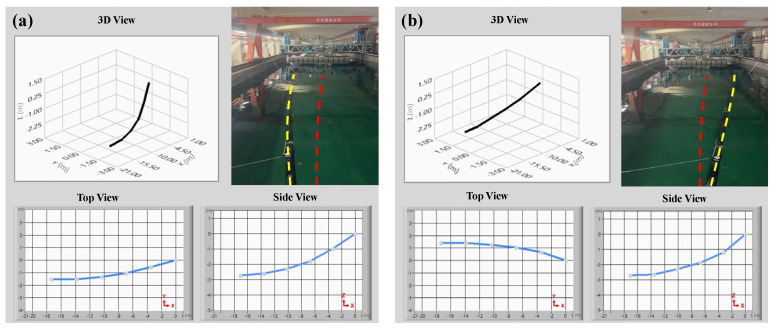
(**a**) Seawater pipeline deflected 1.5 m to the left; (**b**) Seawater pipeline deflected 1.5 m to the right. The red dashed line represents the initial position of the pipeline, and the yellow dashed line represents the actual deflected position.

During the deep-seawater pipeline posture experiments, posture sensing devices were affixed at 3.5 m intervals along the intake pipeline. Array-type pipeline posture sensing modules were used to collect the seawater pipeline’s motion posture signals, which were transmitted to a human–machine interface system. The pipeline posture estimation algorithm was used to calculate the roll, pitch, and yaw during seawater pipeline movement. These posture signals were used to generate the top, side, and 3D views displayed in the human–machine interface, providing a clear and intuitive understanding of seawater pipeline bending or buckling. This validates the accuracy of the pipeline posture sensing modules and estimation algorithms. Figure 9 shows that during the pipeline posture experiments in the towing tank, the pipeline’s outlet end was positioned above the floating platform; the inlet end was bound with ballast blocks to allow it to naturally sink to the tank floor. Waves with a height of approximately 0.5 m were generated by the wave maker to observe changes in pipeline posture. The side view in Figure 9 shows that when the pipeline sank to the tank floor, the human–machine interface illustrates the pipeline resting at a water depth of 3.3 m. Comparatively, a lateral shift of approximately 1.3 m to the left side at the pipeline’s end was produced by the wave’s effect. Figure 10a shows that the pipeline was deflected 1.5 m to the left, producing a leftward bend. The top view of the human–machine interface shows that the pipeline had a lateral shift of approximately 1.5 m to the left. The side view shows that the inlet end of the pipeline was positioned at a water depth of 2.7 m; the outlet end was positioned above the floating platform. Figure 10b shows that the pipeline was deflected 1.5 m to the right, producing a rightward bend. The top view of the human–machine interface shows that the pipeline had a lateral shift of approximately 1.5 m to the right. The side view shows that the inlet end of the pipeline was positioned at a water depth of 2.8 m; the outlet end was positioned above the floating platform. Finally, the seawater pipeline posture observation experiments prove that when the pipeline posture changes because of waves, the pipeline posture sensing module can plot the dynamic signals into a 3D posture diagram of the seawater pipeline through the estimation algorithm, aligning with the actual pipeline postures of the experiment. Therefore, the study can accurately reflect the actual postures of the seawater pipeline. This system can help onshore engineers monitor the status of pipelines in real time, providing immediate feedback while handling abnormal situations promptly to carry out pipeline maintenance in advance and reduce losses.

### 3.2. Seawater Pipeline Leakage Experiments

During the seawater pipeline leakage experiments, five sets of leakage detection modules were arranged at 3.5 m intervals along the pipeline and deployed underwater at a depth of 2 m. Employing Hall sensors and model YF-S201, with a flow error of ±10%, the relationship between the output frequency and flow rate is shown in Equation (12):

(12) $$Q=\frac{F\times 60}{CF}$$

where Q is the flow rate (L/min), F is the sensor frequency (Hz), and CF is the calibration coefficient for the Hall sensor, set to 7.5 for the YF-S201 model.

**Figure 11:**
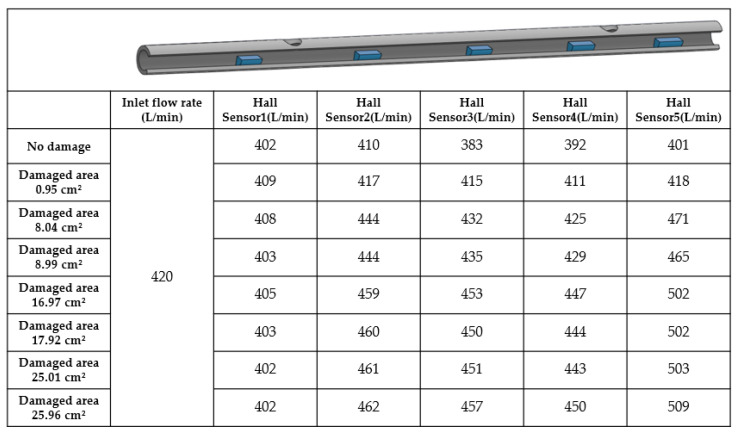
Locations of Pipeline Perforations and Changes in Flow.

In this study, experiments were conducted using a standard flow rate of 420 L/min for seawater pipelines. As water flowed through the pipeline leakage detection modules, the flow rate value was measured; sensor data were transmitted to the human–machine interface. The pipeline leakage algorithm was used to determine the presence, location, and area of perforations. As the perforation area increases, the area estimated from flow rates rises. When damage occurs in the pipeline, both CFD simulations and actual measurements confirm that external pressure exceeding internal pressure causes fluid to enter the pipeline. Hall sensors beside the perforation can detect distinct flow changes under different types of damage as a result. Figure 11 shows that when the pipeline is undamaged, the flow rates recorded by all Hall sensors in the pipeline are nearly identical. The main reason for the slight difference is that when the pipeline is undamaged, the actual flow rate is the flow rate received by the Hall sensor, suggesting no significant difference from the inlet flow rate. The average error value is less than 8%. The error is possibly due to the volume of the Hall sensor blocking the water flow, which causes an unstable flow field. Thus, water flow cannot stably push the gears inside the Hall sensor, leading to a discrepancy between the measured signal on the gears and the theoretical value. Therefore, the flow rate is regarded as identical.

Figure 8 shows how perforation holes of 0.95 cm^2^, 8.04 cm^2^, and 16.97 cm^2^ were designed between Hall Sensor 1 and Hall Sensor 2, and between Hall Sensor 4 and Hall Sensor 5, to investigate the fluid dynamics of the pipeline under damage. Experiment results reveal that the flow rates recorded by Hall Sensor 1 upstream of Hole 1 were nearly unchanged compared to those under the intact condition. However, Hall Sensors 2~4 downstream of Hole 1 exhibit increased flow rate readings as water entered through the perforation at Hole 1. Flow rates at Hall Sensor 5 downstream of Hole 2 significantly increased. As shown in Table 1, perforations were located between Hall Sensors 1 and 2, and between Hall Sensors 4 and 5. In contrast, the pipeline between Hall Sensors 2 and 4 showed no significant change or a decreasing trend in flow rate, indicating the absence of perforations. Therefore, the experiment affirms that differences in flow rates detected by Hall sensors can be used to determine the perforation locations.

**Figure 12:**
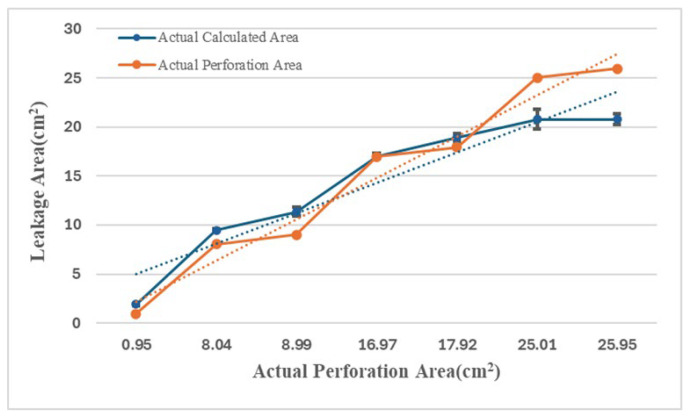
Comparing Estimated and Actual Perforation Areas with Standard Deviation Bars. The dotted line represents the trend line.

**Figure 13:**
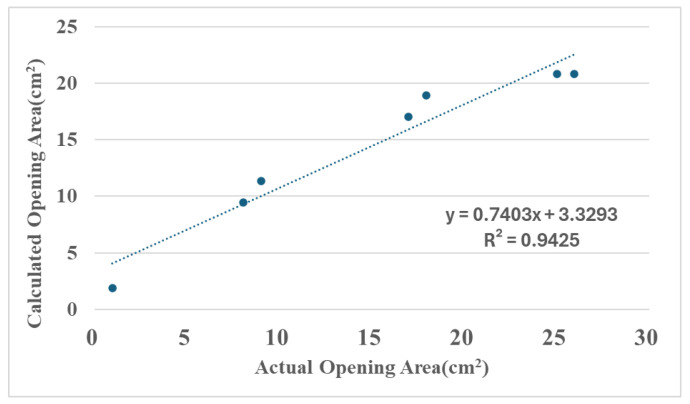
X-Y Distribution of Estimated and Actual Damaged Area Measurements. The dotted line represents the trend line.

As shown in Table 2 and Figure 12, the flow difference is obtained from the disparity between downstream and upstream Hall sensors. The obtained difference is then used to estimate the damaged area through the relationship illustrated in Figure 7. The table shows that particularly at the minimum damage area (0.95 cm^2^), the percentage error in the calculated area reaches as high as 99%, suggesting that the algorithm has low accuracy. However, the errors are due to the limitations of the experimental tools because they primarily stem from the measurement error range of the Hall sensors. When the actual flow variation (7.5 L/min) is minimal and approaches the sensor’s error margin, any slight deviation may be magnified in percentage calculations. As the damage area progressively increases, the flow increment far exceeds the sensor error range. Under this condition, the accuracy of the calculated results may significantly improve. For instance, at a damage area of 16.97 cm^2^, the percentage error may even drop to 0%. Moreover, when the damage area reaches 25.95 cm^2^, the area calculation error is also maintained within 5.16 cm^2^, demonstrating the model’s stability during significant flow variations. This validates that the computation model proposed in this study can provide a valuable prediction under experimental constraints. Figure 13 shows the X-Y distribution of estimated versus actual damage areas, with a trend line R^2^ value of 0.9425. This positive correlation indicator clearly indicates the strong linear relationship between the two datasets, and further illustrates the critical physical inference that an increase in the damage area leads to a greater flow increment. These findings confirm the model’s effectiveness and practical value.

**Figure 14:**
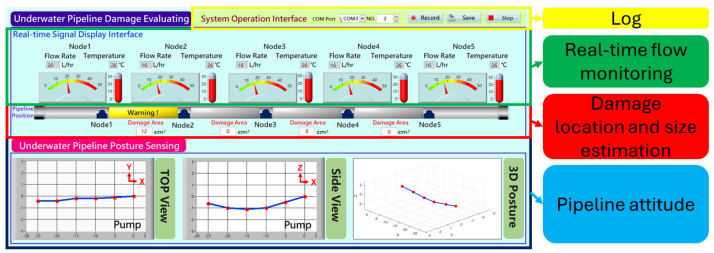
Schematic diagram of the remote human–machine interface.

To achieve effective and timely monitoring of pipeline dynamics, this study developed a human–machine interface, as shown in Figure 14. In the pipeline posture section, the top view and side view of the pipeline can be observed on the human–machine interface. These views are drawn by calculating the pipeline posture signals through the pipeline posture sensing module. Through the 3D view, abnormalities such as bending or buckling of the underwater pipeline can be determined more intuitively. In the pipeline leakage real-time detection section, the instrument can display the flow rates measured by each Hall sensor at corresponding pipeline locations. Data variations between adjacent flow meters can be used for the real-time estimation of perforation areas and locations. Additionally, the logbook function in the upper right corner of the interface records the system’s real-time operational status and alert events, providing an important basis for subsequent data analysis and abnormality tracking.

## 4. Conclusions

This study developed an underwater pipeline monitoring system, which integrates pipeline posture sensing modules with pipeline leakage detection modules to achieve real-time, autonomous, and continuous subsea monitoring capabilities. Using inertial and Hall flow sensors, combined with signal algorithms, the proposed system adopts a configuration design in arrays and utilizes a CAN Bus communication architecture to achieve the effective detection of pipeline posture changes and flow anomalies. Underwater experiments confirmed that the proposed system can accurately capture real-time postures and damage locations of the water intake pipeline. By combining these data with local flow variations, damage areas can be estimated. Experimental results indicate that in cases of minor damage with small flow variations, percentage errors in calculations are elevated due to the inherent error range of the sensors. However, with large damage areas (ranging from 8.04 cm^2^ to 25.96 cm^2^), the estimation results of the proposed system are very close to the actual area trends (R^2^ = 0.9425), and the area error is maintained within 5.16 cm^2^ (error percentages ranging from −20% to 26%). This proves that the system can offer rapid preliminary estimates of damage area under substantial flow changes. The findings of this study can be used to effectively enhance the management efficiency of underwater pipelines, strengthen the timeliness of maintenance work, and significantly reduce the risk of water supply interruptions caused by damage to pipelines by enabling precise location and quantification of damage severity.

## Figures and Tables

**Table 1:** Performance Comparison of Pipeline Monitoring Technologies.

Technology Name	Advantages	Disadvantages	Accuracy
Fiber Optic Sensor (FOS)	High precision and low loss during long-distance transmission Compact and non-invasive Resistant to electromagnetic interference Capable of detecting various types of defects	Brittle, requires permanent installation Costly signal analysis system Requires great signal processing Prone to pressure failure	FBG sensors can enhance accuracy in leak detection, particularly when supplemented by support vector machine (SVM) classifiers, with spatial resolutions typically ranging from 0.01 to 1 m.
Magnetic Flux Leakage (MFL) testing	Simultaneously detects internal and external defects Non-contact testing A well-established technology.	Difficult to quantify the measured values Time-varying impurities may significantly affect the results Highly dependent on operator’s experience Requires operation near pipeline	Quantifying measured values remains challenging. Dipole models cannot stably predict the correct amplitude for specific defect sizes.
Ultrasonic Testing (UT)	Capable of inspecting both internal and external defects High resolution and accuracy, and can provide detailed information on defect volumes and shapes	Requires coupling agents and surface cleanliness Highly dependent on operator experience Increased signal noise due to environmental noise High pressure and temperature affect signal	With high resolution and accuracy, defect can be positioned within approximately ±0.01 m. However, its sensitivity is lower for small holes or cracks.
Electrical Field Mapping (EFM)	Capable of detecting, classifying, and locating corrosion or erosion both internally and externally within the pipe wall Capable of continuous online measurement Wide operational temperature range	Damage estimation based on empirical formulas may incur significant potential errors Conductive deposits may lead to erroneous readings Biofouling and sediment interfere with electrodes	Damage estimation based on empirical formulas may incur significant potential errors. Finite element simulations are used to determine the optimal electrode configuration for mapping pipeline wall defects.
Radiography	Capable of inspecting both internal and external defects Applicable to most underwater pipeline materials Does not require direct contact with the pipeline	Harmful to nearby humans and animals Slow speed; highly dependent on operator experience Significant challenges in offshore operations Requires stronger energy	Capable of detecting defects as small as 0.5 mm in diameter, and can provide highly accurate size and shape information.
Array-based Hall Sensors (used in this study)	Pressure-resistant Easy integration and maintenance Low power consumption, and suitable for prolonged measurements User-friendly, requiring little training	Damage localization resolution depends on the spacing of the array configuration, typically in meters Poor accuracy in predicting damage size Cannot determine the type of damage	In experimental scenarios with significant damage areas (ranging from 8.04 cm^2^ to 25.96 cm^2^), the area error is maintained within 5.16 cm^2^.

**Table 2:** Comparison of Experimental Data and Calculated Results for Flow Increase with Different Damage Areas.

Damage Area (cm^2^)	Flow Difference ± Std Dev (L/min)	Calculated Area ± Std Dev (cm^2^)	Area Error (cm^2^)	Error
0.95	7.5 ± 0.5	1.89 ± 0.13	0.94	99%
8.04	36 ± 0.5	9.45 ± 0.14	1.41	18%
8.99	41.5 ± 0.87	11.34 ± 0.47	2.35	26%
16.97	54.5 ± 0.5	17.01 ± 0.27	0.04	0%
17.92	57.5 ± 0.5	18.9 ± 0.39	0.98	5%
25.01	59.5 ± 0.87	20.79 ± 0.97	−4.22	−17%
25.96	59.5 ± 0.5	20.79 ± 0.58	−5.16	−20%

## Data Availability

Data are contained within the article.
